# The Phenotypical Characterization of Dual-Nature Hybrid Cells in Uveal Melanoma

**DOI:** 10.3390/cancers16183231

**Published:** 2024-09-22

**Authors:** Emily Marcotte, Alicia Goyeneche, Mohamed Abdouh, Julia Valdemarin Burnier, Miguel Noel Burnier

**Affiliations:** 1 Cancer Research Program, Research Institute of the McGill University Health Centre, Montreal, QC H4A 3J1, Canada; alicia.goyeneche@affiliate.mcgill.ca (A.G.); mohamed.abdouh@muhc.mcgill.ca (M.A.); julia.burnier@mcgill.ca (J.V.B.); miguel.burnier@mcgill.ca (M.N.B.J.); 2 The MUHC–McGill University Ocular Pathology & Translational Research Laboratory, McGill University, Montreal, QC H4A 3J1, Canada; 3 Experimental Pathology Unit, Department of Pathology, McGill University, Montreal, QC H3A 2B4, Canada; 4 Gerald Bronfman Department of Oncology, McGill University, Montreal, QC H3A 3T2, Canada

**Keywords:** uveal melanoma, dual-nature hybrid cells, circulating hybrid cells, primary tumors, immunophenotyping, cell co-culture, cell–cell fusion

## Abstract

**Simple Summary:**

Uveal melanoma (UM) is a rare type of cancer of the eye that develops from a cell type called melanocytes, which are present in the uveal tract. These patients have a high risk of liver metastasis, usually many years after the initial diagnosis, which is life threatening. We discovered a population of cells in the primary tumor called dual-nature hybrid cells (DNCs) similar to previously discovered cells in the blood (i.e., circulating hybrid cells, CHCs) with shared characteristics of both cancer cells and white blood cells. In this paper, we determined the types of white blood cells capable of participating in the formation of a hybrid cell with a cancer cell, and we re-created these DNCs in a cell culture. The study of the cells of this tumor, particularly DNCs, is crucial to better understanding dissemination and uncovering a possible target for treatment of this systemic disease.

**Abstract:**

Background: Metastasis, occurring years after primary diagnosis, represents a poor prognosis in uveal melanoma (UM)-affected individuals. The nature of cells involved in this process is under debate. Circulating hybrid cells that have combined tumor and immune cell features found in blood were predictive of metastasis and may correspond to dual-nature cells (DNC) in the primary tumor. Herein, we sought to determine the presence of DNCs in primary UM tumors, the cell types involved in their genesis, and their ability to be formed in vitro. Methods: UM lesions (n = 38) were immunolabeled with HMB45 in combination with immune-cell-specific antibodies. In parallel, we co-cultured UM cells and peripheral blood mononuclear cells (PBMCs) to analyze DNC formation. Results: HMB45^+^/CD45^+^ DNCs were present in 90% (26/29) of the tumors, HMB45^+^/CD8^+^ DNCs were present in 93% (26/28), and HMB45^+^/CD68^+^ DNCs were present in 71% (17/24). DNCs formed with CD8^+^ and CD68^+^ cells were positively correlated to the infiltration of their respective immune cells. Notably, UM cells were prone to hybridize with PBMCs in vitro. Conclusions: This phenotypical characterization of DNCs in UM demonstrates that CD8^+^ T-cells and macrophages are capable of DNC formation, and they are important for better understanding metastatic dissemination, thus paving the path towards novel therapeutic avenues.

## 1. Introduction

Uveal melanoma (UM) is classed as a rare disease, but it remains the most prevalent primary intraocular malignant tumor found amongst adults [1]. The incidence is approximately 2–10 patients per million depending on the region, with the vast majority being light-skinned [1,2,3]. This malignancy typically presents in a unilateral manner and can be located within the choroid, ciliary body, or iris [4,5]. Treatments have improved as certain options, such as radiotherapy, have replaced enucleation in most cases, ultimately enhancing patients’ quality of life through the preservation of the eye [6]. Despite this, survival has not improved, as metastasis still leads to death in over 40% of patients more than 15 years after the initial diagnosis [1,7,8,9]. These metastases are due to hematogenous dissemination occurring preferentially in the liver, which is affected in more than 90% of metastatic cases [10,11]. 

In order to stratify the prognosis of UM, clinical and histopathological prognostic factors, such as cell type, tumor size, the presence of vascular loops, mitotic figures, scleral invasion, and infiltrative inflammatory cells, have been established [12,13]. Patients with a predominantly epithelioid cell type, larger tumors, vascular loops, high mitotic counts, scleral invasion, and the presence of inflammatory cells have a worse prognosis (i.e., a higher incidence of metastasis and death). In contrast, patients with a predominantly spindle cell type, smaller tumors, decreased or no vascular involvement, lower or no mitotic counts, no scleral invasion, and no inflammatory cells have a better prognosis [12,13,14,15,16,17,18,19,20,21,22,23]. Moreover, the loss of the heterozygosity of chromosome 3 and the gain of the chromosome 8q arm were reported as the most relevant chromosomal abnormalities, and they are associated with poor prognosis [24]. 

The initiation and progression to metastatic disease in UM still raises many unanswered questions. The most common mutations in UM are in GNAQ or GNA11. Some research has pointed to initiation involving Hippo signaling through the YAP/TAZ pathway [25,26]. The inhibition of the Hippo tumor-suppressor pathway through YAP has been shown to be correlated to GNAQ/11 status as well as being important for tumor initiation in UM [25,26]. In fact, YAP inhibition with verteporfin has been shown to block tumorigenesis [26]. In addition, mitogen-activated protein kinase (MAPK), a potential downstream target in UM, has been demonstrated to have variable activation in tumors with GNAQ/11 mutation, contributing not only to tumor heterogeneity, which complicates potential treatment options, but also therapy resistance, as seen in other cancers [27,28]. 

During the metastatic cascade, tumor cells detach from the primary tumor, invade the surrounding tissue, intravasate, and stay alive in circulation before extravasation and successful colonization in secondary organs. Non-invasive procedures, such as liquid biopsy, are being investigated to find new prognostic factors and to track the natural course of the disease [29]. This is a technique in which bodily fluids are evaluated for tumor DNA, extracellular tumor vesicles, and/or circulating tumor cells (CTCs) [29]. Previously, our laboratory and others reported on the presence of CTCs in the peripheral blood of almost all patients with UM regardless of treatment, and they were thought to be predictors of poor prognosis and thus predictors of the development of metastasis [30,31,32]. These cells home at metastatic sites under a dormancy state, as we reported in a previous animal model study, which may be why micro- and macrometastases take place years after primary disease diagnosis [33].

The discovery of circulating hybrid cells (CHCs) emerged, and they were described as cells with dual-nature characteristics of both tumor cells and leukocytes [34]. These cells were found in the circulation of many epithelial and non-epithelial cancers, including UM [35]. In the context of UM, this novel cell type rivaled the hypotheses of CTCs by only finding CTCs in the blood of less than 60% of patients and finding CHCs in all patients and in greater quantities than their counterparts, making them a better predictor of metastasis and a vital component of better understanding UM progression [34]. In addition, the generation of hybrids and their presence in circulation and metastatic sites in cancer support the hypothesis that cell–cell fusion might be a mechanism associated with metastatic dissemination [36,37].

Dual-nature hybrid cells (DNCs), which are thought to correspond to CHCs found in the blood, have only been recently evaluated in the primary tumor [38]. Based on these recent findings of hybrid cells in UM patients, we aimed to uncover the presence of DNCs in a larger sample size of primary UM lesions and determine the leukocytic cell types involved in their formation. In addition, the capacity of hybrid cell formation was also tested in vitro by co-incubating UM cells with peripheral blood mononuclear cells (PBMCs). Overall, DNCs were detected in the majority of primary UM samples displaying a dual phenotype of UM cells (i.e., HMB45) and that of leukocytes (i.e., CD45), including, mainly cytotoxic T cells and macrophages (i.e., CD8^+^, and CD68^+^, respectively). In vitro co-culture experiments showed that UM cells and PBMCs are inclined to hybridize. These findings support the involvement of heterotypic hybrid cells in primary solid tumors and the capacity of UM cells to fuse with normal leucocytes. This offers a step towards a better understanding of the metastatic process for the development of new UM therapies. 

## 2. Materials and Methods

### 2.1. Patient Specimens

Thirty-eight specimens of UMs were collected from the MUHC–McGill University Ocular Pathology & Translational Research Laboratory between 1980 and 2020. Demographic information pertaining to age and sex were obtained when available. 

For morphology, histopathological type was classified according to the revised Callender classification of epithelioid, spindle, or mixed cell type [39]. Mixed-cell tumors were then further classified according to their predominant cell type. Vascular loops, mitotic figures, and scleral invasion were evaluated through hematoxylin and eosin (H&E) analysis. Characteristics were validated using the original pathology report when available (Table 1).

### 2.2. Histological Staining and Immunohistochemical (IHC) and Immunofluorescence (IF) Analyses 

Formalin-fixed, paraffin-embedded sections (4 µm thick) from enucleated globes were deparaffinized and rehydrated for subsequent H&E staining and immunolabeling. 

A standard H&E procedure was performed using the automated stainer with a coverslip applicator from Leica (Leica CV5030 and ST5020 Stainer, Leica Biosystems, Nussloch, Germany).

For IHC analysis, we performed both single and dual labeling using the Discovery Ultra system from Roche (Graz, Austria). Following antigen retrieval treatment using Tris-EDTA for 36 min (32 min for CD3 staining), the sections were incubated for 24 min at 37 °C with anti-HMB45 antibody (Ab) at 1/45 dilution (M0634, Dako, Hilden, Germany), prediluted anti-CD45 Ab (760-4279, Roche), prediluted anti-macrophage CD68 Ab (760-2931, Roche), prediluted anti-CD3 Ab (790-4341, Roche), or anti-MelanA Ab at 1/40 dilution (NCL-L-MelanA, Leica). After washing, slides were incubated with secondary Ab for 20 min (OmniMap anti-rabbit HRP 760-4311, Roche for CD3), followed by the detection Kit UltraView Universal DAB (760-159, Roche) or the Discovery Red HRP kit (760-260, Roche in the case of CD3 staining). Slides were then counterstained with the hematoxylin, dehydrated, and cleared, and a coverslip was applied. 

For dual IHC labeling, sections were incubated for 24 min at 37 °C with anti-HMB45 Ab, washed, and incubated with the secondary Ab, OmniMap anti-Mouse (Mse) HRP (760-4310, Roche) at RT for 24min, followed by the detection Kit Discovery Purple (RUO, 760-229, Roche). After Ab denaturation, sections were incubated for 24 min at 37 °C with prediluted anti-CD45 Ab, prediluted anti-CD8 Ab (790-4460, Roche), or prediluted anti-CD68 Ab. They were then incubated with the secondary Ab, OmniMap anti-Mse HRP (760-4310, Roche), at RT for 20 min, followed by the detection kit Discovery Teal (RUO, 760-247, Roche) or the detection Kit Discovery Green (RUO, 760-271, Roche) for some of the CD68 double-labeled slides. Slides were then counterstained with the hematoxylin, dehydrated, and cleared, and a coverslip was applied. 

For IF staining, slides were incubated for 24 min at 37 °C with anti-HMB45, washed, and. incubated with the secondary Ab, OmniMap anti-Mse HRP coupled with Fluorescein isothiocyanate (FITC; 760-232, Roche) at RT for 24 min. After Ab denaturation, sections were incubated for 24 min at 37 °C with prediluted anti-CD8 Ab. Then, the slides were incubated with the secondary Ab, OmniMap anti-Rb HRP (760-4311, Roche) coupled with Rhodamine (760-233, Roche), at RT for 20 min. Slides were then counterstained with the DAPI and rinsed, and a coverslip was applied with Permafluor mounting media. 

Slides from all labeling processes were digitally scanned using a ZEISS AxioScan.Z1 and then analyzed using ZEISS ZEN blue software version 3.5 (Carl ZEISS AG, Oberkochen, Germany).

### 2.3. Cell Culture

#### 2.3.1. Normal Choroidal Melanocyte (NCM) Culture Medium

NCM cells were gifted by Dr. Julia Burnier’s laboratory (McGill University, Montreal). These cells were isolated as described previously from human cadaveric eyes in accordance with a protocol approved by the IRB of the Research Institute of the McGill University Health Centre (RI-MUHC) (IRB #2019-5314) [40]. Cells were maintained in Melanocyte Growth Medium M2 (C-24300, PromoCell, Heidelberg, Germany). Cells were incubated in a humidified incubator at 37 °C under 5% CO_2_. Cell viability was assessed through trypan blue exclusion assay. The number of cells was calculated using the TC20 Automated cell counter (Bio-Rad, Mississauga, ON, Canada). 

#### 2.3.2. UM Cell Culture Medium

UM 92.1 cells were gifted by Dr. Martine Jager (Leiden University, The Netherlands) [41]. MEL270 and OMM2.5 cells were gifted by Dr. Vanessa Morales (University of Tennessee). Cells were maintained in RPMI 1640 medium supplemented with 10% fetal bovine serum (FBS), 10 mM HEPES, 2 mM of Glutagro supplement, 1 mM of sodium Pyruvate, 100 U/mL of penicillin, 100 μg/mL of streptomycin (all from CorningTM, Lauderdale, FL, USA), and 10 μg/mL of insulin (Millipore-SIGMA, Oakville, ON, Canada). Cells were incubated in a humidified incubator at 37 °C under 5% CO_2_. Cell viability was assessed through trypan blue exclusion assay. The number of cells was calculated using the TC20 Automated cell counter (Bio-Rad). 

#### 2.3.3. Isolation of Peripheral Blood Mononuclear Cells (PBMCs) 

Blood (10 mL) was collected from healthy individuals at the McGill Academic Eye Clinic in accordance with an approved ethics protocol (#2018-4187) by the Review Ethic Board of the RI-MUHC. The blood was diluted 1/1 (*v/v*) with cold phosphate-buffered saline (PBS) + 1 mM EDTA + 2% FBS, and density gradient centrifugation was used to isolate mononuclear cells using Lymphoprep solution and Sepmate tubes (StemCell Technologies, Vancouver, BC, Canada). Briefly, Lymphoprep was layered in the Sepmate tube insert, and the diluted blood was added slowly at the top of the insert. Samples were centrifuged at 1200 g for 10 min without the brake. The top layer was poured into a new tube and centrifuged at 300 g for 8 min. The supernatant was removed, and the pellet was washed twice with PBS-2% FBS-1mM EDTA at 300 g for 8 min. The pellet was resuspended in 10 mL of RPMI medium without serum, and PBMCs were counted and evaluated for viability.

#### 2.3.4. UM Cells, PBMC Labeling, and Co-Culture 

UM 92.1 cell mitochondria were tagged with Mitotracker M7514 (Invitrogen, San Diego, CA, USA). Cells were seeded the day before; therefore, the staining of the mitochondria was performed on adherent cells according to the manufacturer’s instructions. Excess staining was removed, and the culture was washed twice with fresh media.

The membrane of PBMCs was stained with the PKH26 red fluorescent cell linker (Millipore-SIGMA) following the manufacturer’s protocol. PBMCs were spun at 300 g for 10 min at room temperature (RT), and the pellet was resuspended in complete medium and transferred to a new tube. PBMCs were washed another 2 times at 300 g for 5 min at RT with complete medium to ensure the removal of unbound dye. Finally, the pellet was resuspended in complete medium, and PBMCs were counted and evaluated for viability.

Mitotracker-labeled 92.1 cells were trypsinized, counted, and combined with PKH26-labeled PBMCs. A total of 0.24 × 10^6^ cells in a 24-well plate with different combinations between UM and PBMCs (UM cells/PBMCs: 1/10; 1/5; 1/3; 1/1; 3/1; and 5/1) were seeded, and the interactions between PBMCs and cancer cells were followed every two hours using the IncuCyte^®^ S3 Live-Cell Analysis System. A standard scan type with 10 × magnification was applied. Excitation/emission of 490/516 nm for the mitochondria tracker and 551/567 nm for the PBMCs’ membrane staining were selected to identify fusion events between cancer cells and PBMCs. 

On the other hand, UM 92.1 cells (0.6 × 10^6^) were seeded into glass-bottom dishes (Mat Tek) for 24 h and stained with the Mitotracker. An equal number of PKH26-labeled PBMCs (0.6 × 10^6^) was combined with Mitotracker-labeled adherent UM cells. Interactions between 92.1 cells and PBMCs were followed by capturing microscopic images for 30 s every 2.0 min for a total time of 90 min. Scanning was performed under Zeiss LSM780-NLO laser scanning confocal with IR-OPO lasers. The objective used was EC Plan-neofluor 10 × /0.3 na. Laser red 551/567 and green 490/516 were used for data acquisition. At each phase, green and red images were recorded, and different movies were created to follow cell–cell interactions.

In a parallel set of experiments, UM cells and PBMCs were stained with PKH26 and PKH67 dyes, respectively. Cells were mixed at different ratios (UM cells/PBMCs: 1/3, 1/5, and 1/10) and incubated for 24 h in 8-well chamber slides. The slides were washed with PBS and mounted in DAPI-containing medium (Vector, Malvern, PA, USA). Images were acquired using an LSM780 confocal microscope (Zeiss, Malvern, PA, USA).

### 2.4. Phenotypic Determination of Co-Cultured NCM Cells and PBMCs, as Well as UM Cells and PBMCs

NCM cells and PBMCs were co-cultured in tissue culture slide flasks (177,453, Lab-Tek Flaskette Chamber Slide) for 3 days at different ratios (1:1, 2:1, 1:2). Adherent cells were fixed in 2% paraformaldehyde for 20 min at RT. The fixative was removed, and cells were washed twice with PBS. After antigen-retrieval treatment (EDTA buffer, 8 min), the slides were incubated for 24 min at 37 °C with anti-CD45 (760-4279, Roche). Next, secondary Ab incubation was performed with OmniMap anti-Mse HRP (760-4310, Roche) at RT for 20 min followed by the detection Kit Discovery purple (760-247). After Ab denaturation, the slides were incubated for 24 min at 37 °C with anti-HMB45 Ab at a dilution of 1/45 (M0634, Dako) and exposed to the secondary Ab OmniMap anti-Mse HRP (760-4310, Roche) at RT for 20 min, followed by the detection Kit Discovery Green kit (760-271, Roche). Slides were then counterstained with the hematoxylin, dehydrated, and cleared, and a coverslip was applied. 

After 7 days of co-culture between 92.1 UM cells and PBMCs, adherent cells were trypsinized and counted. Collected cells (0.3 × 10^5^) were replated in 8-well chamber slides. After cell adherence (24 h), cells were fixed in 4% paraformaldehyde for 25 min at RT. The fixative was removed, and cells were washed twice with PBS. Slides were incubated for 24 min at 37 °C with anti-HMB45 Ab at a dilution of 1/45 (M0634, Dako), washed, and exposed to the secondary Ab, OmniMap anti-Mse Horseradish peroxidase (HRP) (760-4310, Ventana-Roche) at RT for 24 min, followed by the detection Kit Discovery Purple (760-229, Ventana-Roche). After Ab denaturation, the slides were incubated for 24 min at 37 °C with prediluted anti-CD45 Ab (760-4279, Ventana-Roche), washed, and exposed to the same secondary Ab, followed by the detection kit Discovery Teal kit (RUO, 760-247, Roche). The slides were then counterstained with the hematoxylin, dehydrated, and cleared, and a coverslip was applied.

### 2.5. Statistical Analysis 

All statistical analyses were performed using GraphPad Prism (GraphPad Prism version 9.5.0, GraphPad Software Inc., La Jolla, CA, USA). The Spearman’s rank test was used for the correlation analysis between infiltrating inflammatory cells (i.e., CD8^+^ T-cells and CD68^+^ macrophages) and the presence of DNCs coupled with a linear regression. A Chi-squared test in conjunction with the Fisher’s exact test was performed to test for a relationship between the presence of high-risk factors (HRFs) and the presence of DNCs.

## 3. Results

### 3.1. Immunohistochemical (IHC) Analysis of UM Tumors 

From the 38 patients included in this study, demographic information pertaining to sex and age at the time of diagnosis were available for 36 and 34 of the patients, respectively (Table 1). Of the 36 patients, 56% (n = 20) were male and 44% (n = 16) were female. The mean age at diagnosis was 65.3 (±13.6) years (Table 1). All tumors expressed diffuse staining of UM-specific markers, HMB45 and MelanA (Figure S1).

In parallel, histopathological examination following H&E staining revealed that analyzed tumors exhibited HRFs associated with poor patient prognosis (i.e., large lesions (53%, n = 20), which were predominantly of the epithelioid cell type (61%, n = 23) and the presence of closed vascular loops (34%, n = 13), mitotic figures (42%, n = 16), and scleral invasion (29%, n = 11) (Figure S2). 

### 3.2. Most UM Tumors Contained DNCs with HMB45^+^/CD45^+^-, HMB45^+^/CD8^+^-, and HMB45^+^/CD68^+^-Dominant Phenotypical Profiles

**Figure 1:**
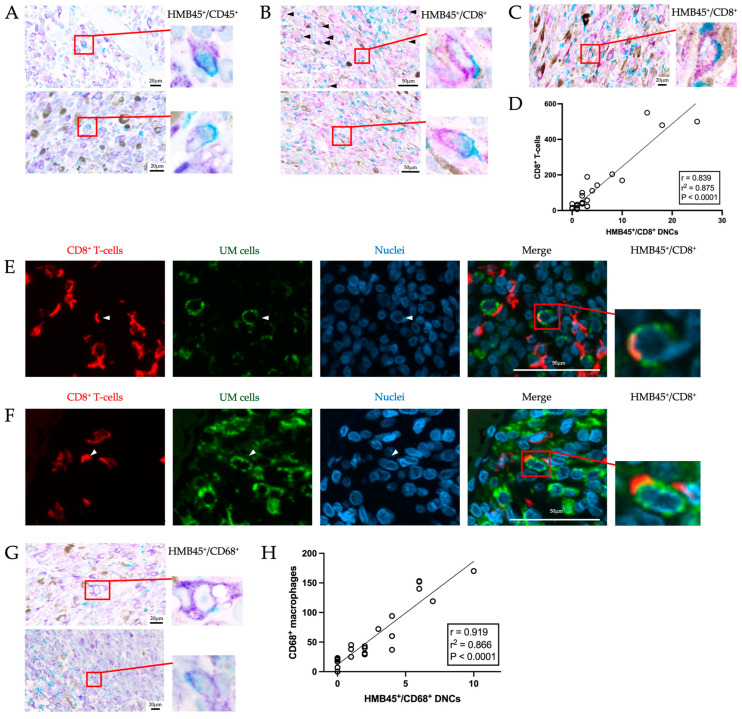
Phenotypic characterization of DNCs in the primary tumor. (**A**) HMB45-positive cells (purple), CD45-positive cells (teal), and HMB45^+^/CD45^+^ DNCs (red boxes) with a higher-power image of the DNCs. (**B**) HMB45-positive cells (purple), CD45-positive cells (teal), and HMB45^+^/CD8^+^ DNCs (black arrowheads and red boxes) with a higher-power image of the DNCs. (**C**) Multinucleated HMB45^+^/CD8^+^ DNC (red box) with a higher-power image of the DNC. (**D**) Spearman correlation of the number of HMB45^+^/CD8^+^ DNCs versus the number of CD8^+^ T-cells with a significant r value (r = 0.839, *p* < 0.0001). The r^2^ value was obtained through linear regression (r^2^ = 0.875). (**E**) Immunofluorescence labeling with CD8 and HMB45 antibodies as well as DAPI (nuclei) showing an HMB45^+^/CD8^+^ DNC (merge; white arrowheads) and its higher-power image. (**F**) Immunofluorescence labeling with CD8 and HMB45 antibodies as well as DAPI (nuclei) showing a multinucleated HMB45^+^/CD8^+^ DNC (merge; white arrowheads) and its higher-power image. (**G**) HMB45-positive cells (purple), CD68-positive cells (teal), and HMB45^+^/CD68^+^ DNCs (red boxes) with a higher-power image of the DNCs. (**H**) Spearman correlation of the number of HMB45^+^/CD68^+^ DNCs versus the number of CD68^+^ macrophages with a significant r value (r = 0.919, *p* < 0.0001). The r^2^ value was obtained through linear regression (r^2^ = 0.866).

To test for the presence of hybrid cells in the primary UM tumor, IHC double labeling with the melanocytic marker, HMB45, and the pan-leukocytic marker, CD45, was performed on all 38 tumors. Of the 38 tumors, 9 were excluded due to the stain not being present on the slide or due to the tumor being too necrotic. Cells were considered to be HMB45^+^/CD45^+^ DNCs when the membrane and/or the cytoplasmic staining demonstrated positive labeling for both markers, and tumors were categorized as positive when at least one DNC was scored (Figure 1A). In contrast, tumors in which the cells had only one marker but were in close proximity to another cell or in which distinct cells were touching with unique nuclei and without any cytoplasmic involvement were considered to be negative. Using these criteria, we detected HMB45^+^/CD45^+^ DNCs in 90% (n = 26/29) of the tumors (Figure 1A). When looking at the hybrid cells, they all possessed a singular nucleus and one continuous cell membrane with areas of overlap in positivity between both markers. The melanosome marker, HMB45, displayed a more granular staining appearance in comparison to CD45, which displayed a much smoother or linear staining. Due to high variability in the overall morphology of the cancer and the leukocytic cells, no conformity in size or shape could be seen amongst the HMB45^+^/CD45^+^ DNCs.

To gain more insight into the identity of immune cells involved in DNC formation, we looked at the expression of immune-cell-specific markers, namely, cytotoxic T-cells and macrophages.

We evaluated the presence of tumor-infiltrating lymphocytes (TILs) in 30 of the primary tumors, including, mainly, T-cells, by using the pan-T-cell marker CD3. All were positive for CD3, with counts ranging from 1 to >100 TILs in 10 high-power fields (HPFs). Only 9 had moderate to high positivity (>50 TILs in 10 HPFs), with the 21 others having low positivity (<50 TILs in 10 HPFs; Figure S3A). 

Despite many tumors having low CD3 positivity, all 38 were double-labeled with HMB45 and CD8, a marker for cytotoxic T-cells. Of the 38 tumors, 10 were removed from the analysis due to there being no stain present on the slide or the majority of the tumor being necrotic. Of the remaining 30 samples, CD8 positivity was seen in 100%, with counts ranging from 7 to >100 cells in 10 HPFs. Moderate to high positivity (>50 TILs in 10 HPFs) was seen in 12 (43%) of the tumors, and low positivity (<50 TILs in 10 HPFs) was seen in the other 16 (57%) tumors (Figure S3B). The presentation of the CD8 T-cells was mainly diffuse, with some multifocal nests. When searching for the presence of HMB45^+^/CD8^+^ DNCs, we used the same criteria as those used for HMB45^+^/CD45^+^ DNC evaluation. HMB45^+^/CD8^+^ DNCs were identified in 93% (n = 26/28) of the tumors (Figure 1B). These HMB45^+^/CD8^+^ DNCs had a single nucleus, and although they were all different in size, they demonstrated a similar pattern where the HMB45 marker was positive in most of the perinuclear region, whereas CD8 was positive only on a small portion (Figure 1B). Although the majority of HMB45^+^/CD8^+^ DNCs possessed a singular nucleus, multinucleated cells with a positive membrane and/or cytoplasmic staining could also be identified (Figure 1C). To look for a relationship between the level of TILs and the presence of DNCs, a Spearman rank test was performed. This analysis revealed a significant positive correlation between the number of CD8^+^ T-cells and the number of HMB45^+^/CD8^+^ hybrid cells counted in 10 HPFs (r = 0.839, linear regression r^2^ = 0.875, *p* < 0.0001; Figure 1D). Following a Chi-squared test and Fisher’s exact test, no statistically significant relationship could be observed between the presence of HMB45^+^/CD8^+^ DNCs and the HRFs present in the primary tumors (Table 2).

Next, five tumors with high numbers of HMB45^+^/CD8^+^ DNCs were labeled with immunofluorescent (IF) markers to verify the co-expression of both of these markers in a single cell. Notably, we observed cells with positivity for both the CD8 and the HMB45 marker (Figure 1E). In addition, from these IF-labeled tumors, a subset of HMB45^+^/CD8^+^ DNCs could be identified as bi- or multinucleated cells, which also demonstrated the co-localization of HMB45 and CD8 (Figure 1F, Figure S4). 

To test tumor-associated macrophages (TAMs) as a potential partner in the formation of DNCs, only 35 of the 38 tumors were double-labeled with HMB45 and CD68 due to some of the blocks being exhausted. Criteria to consider cells to be HMB45^+^/CD68^+^ DNC were as stated above for HMB45^+^/CD45^+^ DNCs. Of the 35 slides, 11 were removed from the analysis due to there being no stain present on the slide or if the majority of the tumor was necrotic. HMB45^+^/CD68^+^ DNCs were present in 71% (n = 17/24) of the tumors (Figure 1G). When looking at the presence of TAMs in the primary tumor, 1 tumor had no macrophages in 10 HPFs, moderate to high positivity (>50 TAMs in 10 HPFs) was seen in 8 tumors (33%), and low positivity (<50 TAMs in 10 HPFs) was seen in 16 tumors (67%) (Figure S3C). To look for a relationship between the level of TAMs and the presence of DNCs, a Spearman rank test was performed. This analysis revealed a significant and positive correlation between the number of CD68^+^ macrophages and the number of HMB45^+^/CD68^+^ hybrid cells counted in 10 HPFs (r = 0.919, linear regression r^2^ = 0.866, *p* < 0.0001; Figure 1H). Following a Chi-squared test, a statistically significant relationship could be observed between the presence of HMB45^+^/CD68^+^ DNCs and the cell type; however, this same significance was not observed after Fisher’s exact test (Table 2). The presence of these hybrid cells showed no statistically significant relationship to any of the other HRFs using both tests.

### 3.3. NCM Cells and PBMCs Are Not Prone to Fusing In Vitro to Form DNCs

To begin, a co-culture between NCM cells and PBMCs was performed to test whether normal melanocytes possess the ability to spontaneously form bi- or multinucleated hybrid cells with normal PBMCs. After 72 h of co-culturing between NCMs and PBMCs, these cells were double-labeled with HMB45 and CD45 antibodies (Figure S5) to search for the presence of any HMB45^+^/CD45^+^ DNCs. We did not observe the presence of DNCs in these co-cultures; however, we did observe interactions between both cell types. Given that we defined the formation of DNCs as a single cell with both markers that is single- or multinucleated, the NCM and PBMC co-cultures did not fit these criteria. Conversely, we did observe a tendency for NCMs to form bi- or multinucleated populations within a cell of NCM origin (single-stained HMB45-positive) in the co-cultures but not in the single cultures (Figure S6). When counting at 40X, approximately 5% of the NCMs appeared to be multinucleated.

### 3.4. UM Cells and PBMCs Are Prone to Fusing In Vitro to Form DNCs

**Figure 2:**
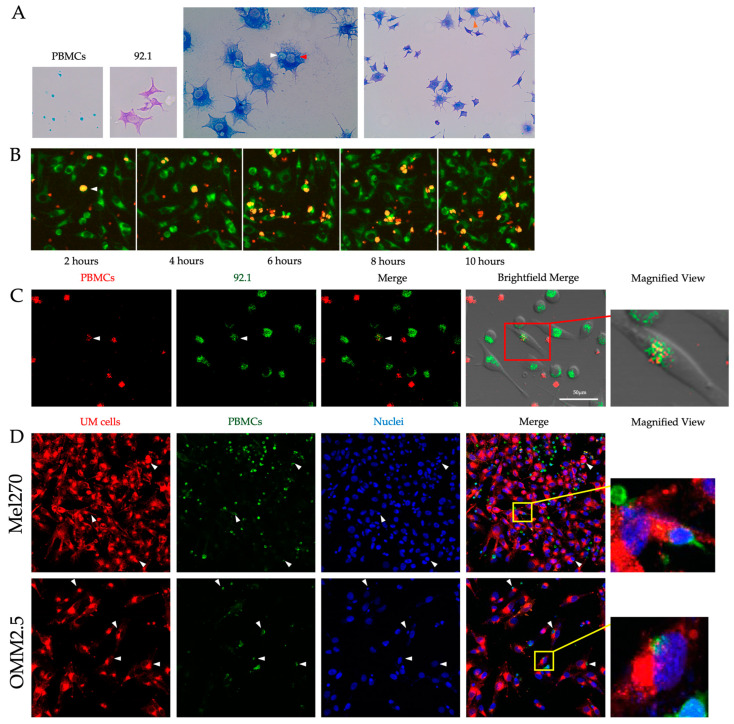
In vitro DNC formation between UM cells and PBMCs. (**A**) PBMCs co-cultured with 92.1 UM cells and immunolabeled with CD45 (teal) and HMB45 (purple), respectively. Multinucleated DNCs with CD45 positivity (white arrowhead) and HMB45 positivity (red arrowhead) imaged at 40× magnification as well as DNCs with CD45 positivity within the cytoplasmic perinuclear region (orange arrowhead) imaged at 20× magnification. (**B**) Real-time live imaging every 2 h under an IncuCyte System of Mitotracker-labeled 92.1 UM cells (green) co-cultured with PKH26-labeled PBMCs (red). Co-localization is interpreted as a positive cell fusion event (DNCs; yellow color, white arrowhead). (**C**) Time-lapse confocal microscopy of PKH26-labeled PBMCs (red) co-cultured with Mitotracker-labeled 92.1 UM cells (green). A cell undergoing fusion (DNC; white arrowhead) can be seen in the merged view coupled to a phase-contrast field view to show the shape of the cells. Higher magnification of the DNC (red box) is shown on the right. (**D**) Confocal microscopy of PBMCs (green; PKH67 dye) co-cultured with Mel270 or OMM2.5 (red; PKH26 dye). Nuclei stained with Dapi (blue). DNCs can be visualized in the merge view (white arrowheads), and a higher magnification of a DNC is shown on the right (yellow boxes).

A co-culture approach was used to test if UM cells are capable of a spontaneous interaction with normal PBMCs to form hybrid cells (DNCs) in cell culture, which may be associated with the process of cell fusion. Following co-culture between 92.1 UM cells and PBMCs, as stated in the Materials and Methods, the cells were double-labeled with HMB45 and CD45 antibodies (Figure 2A). When searching for the presence of HMB45^+^/CD45^+^ DNCs, we found cells with a wide variety of shapes and sizes in addition to finding cells that were multinucleated. DNCs presented cytoplasmic positivity for CD45 in the perinuclear region within what we defined as a cancer cell based on its size and HMB45 positivity (Figure 2A).

Next, we performed real-time live cell imaging using the IncuCyte system by tagging 92.1 UM cells and PBMCs with fluorescent mitochondrial or membrane markers, respectively. The UM cells were adhered, stretched, and identified through the presence of perinuclear green staining, and PBMCs were identified as small, round, red moving cells (Figure 2B). Starting at 2 h following the co-cultures, we observed the formation of fused cells reminiscent of DNCs (as judged by the yellow fluorescence; Figure 2B). Notably, the number of fused cells increased in a time-dependent manner and reached a maximum 6 h post-co-culturing.

To further confirm the spontaneous heterotypic fusion event between these cell types, we subjected 92.1 UM cells and PBMC co-cultures to time-lapse confocal microscopy (Figure 2C). Cancer cells were adhered and stretched within the cell culture plate, whereas PBMCs were constantly moving in the medium. Movies were created with images taken every 2 min to address interactions between cells during the time of incubation. A representative snap-shot image showed that 92.1 UM cells and PBMCs fused to form a DNC-like cell. This heterotypic fusion between the two cell types is seen through the display of a yellow color (Figure 2C). Under the phase-contrast view, the PBMC is shown to be in the plane of the adherent cancer cell and, in particular, in close contact with the mitochondrial marker within the limits of the UM cell, thus confirming the cell fusion event (Figure 2C). 

To continue elucidating the concept of cell fusion in vitro, the Mel270 and OMM2.5 UM cell lines, which were derived from a primary tumor, and its liver metastasis, respectively, were co-cultured with PBMCs for 24 h. Imaging of these cells was performed via confocal microscopy and revealed the presence of a population of fused cells displaying dual-cell membrane labeling (PKH26 and PKH67) (Figure 2D). These cells appeared to have a single nucleus representing what can be interpreted as a DNC cell similar to what was observed in the primary tumors. Altogether, these data provide evidence for UM cell and immune cell fusion in vitro.

## 4. Discussion

**Figure 3:**
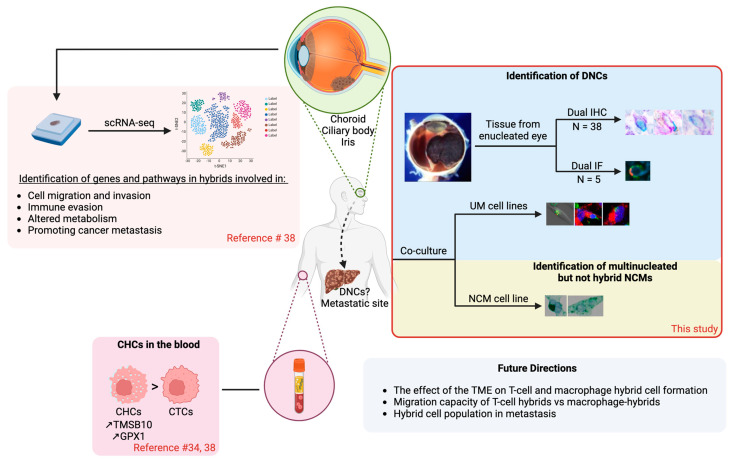
Schematic representation of our study and the relevant literature in relation to hybrid cell formation in UM. A proposed model is shown illustrating the results of NCM interactions and DNC formation in the primary tumor and UM cell lines in our study as well as the relevant hybrid cell literature investigating patient blood and primary samples using single-cell RNA sequencing. GPX1: Glutathione peroxidase 1; TMSB10: Thymosin beta 10. Figure made using BioRender.

Seeding of metastasis in UM was initially thought to be predicted by the dissemination of CTCs in the peripheral blood of patients, as systemic disease could be found as early as the time of the primary diagnosis (i.e., treatment-naïve), which made these cells an important prognostic factor for disease progression [30,31,32,42]. The discovery of hybrid cells in various solid tumors, such as colon, prostate, breast, and intestinal cancers, where these cells are now thought to be a cause of metastasis has led to a shift in research [34,35,43,44]. These CHCs were also identified in the blood of UM patients, where they were studied as a predictor for metastasis [34,35]. Their characterization in patients’ blood samples was based on dual phenotypic features; i.e., melanocytic and leukocytic phenotypes (gp100^+^/CD45^+^) [34]. A small subset of primary tumors (n =1 and n = 3) was found to have hybrid cells positive for CD45 and melanocytic markers (Figure 3) [38]. In our study, we tracked these cells in a larger subset of ocular UM lesions using the anti-melanosome HMB45 and CD45 markers. We identified HMB45^+^/CD45^+^ DNCs in 90% (n = 26/29) of the analyzed specimens (Figure 3). This percentage is very close to what was observed when patients’ blood was analyzed, as hybrids were identified in 100% of the samples [34]. Given that research on CTCs has demonstrated that UM is much more of a systemic disease and that we know hybrid cells are present in the blood, any differences observed between the primary tumor and the blood may be explained by dissemination from the primary site having already taken place, or the evaluation of the primary tumor may represent an underestimation of the true number of DNCs as a result of the number of tissue sections taken from each tumor [30,34]. It is important to note that many of these hybrid cells might not survive the resolution of the multinucleation, causing only those that succeed to be found in the blood, which are potentially capable of propagating, thus leading to a larger population. Interestingly, in UM and other cancers from solid tumors, CHCs were identified in greater numbers in the blood in comparison to CTCs [34,35]. No statistically significant relationship was observed between the presence of HMB45^+^/CD45^+^ DNCs and HRFs. Overall, just like CHCs in the blood, DNCs in the primary tumor may represent a novel and promising prognostic biomarker and target cell in UM.

We found that the main immune cell players involved in DNC formation were macrophages and cytotoxic T-cells (Figure 3). T-cells, which are part of the adaptive immune system, form a class that is subdivided into three subtypes (i.e., T-helper cells, cytotoxic T-cells, and T-regulatory cells) [23]. In general, hot tumors are characterized by high infiltration of non-exhausted T-cells, especially CD8^+^ T-cells, whereas cold tumors lack T-cell infiltration and fail to induce T-cell priming [45]. In UM, T-cell infiltration, particularly CD8^+^ cytotoxic T-cells, has been shown to be an indicator of worse patient prognosis through its correlation with the development of metastasis [12,22,23]. It is known that the most malignant UM tumors have monosomy in chromosome 3 and the absence of the expression of BRCA1-associated protein 1 (BAP1) [46]. BAP1-negative tumors, in particular, have been shown to display an immunosuppressive environment [46]. Furthermore, half of UM tumors display an increased expression of immunomodulatory molecules related to T-cell suppression [46]. This unfavorable immune response causes UM to differ from other solid tumors, including cutaneous melanoma, where the presence of TILs would usually represent a good patient prognosis [23,47]. In our study, we found CD8^+^ T-cells to be present in 100% of the tumors, with variable levels of infiltration, which is in agreement with the literature [48]. Typically, there was diffuse presentation of these cells, with some focal areas of nest formation, which is also in agreement with the literature [22]. In our analyzed cohort (n = 28), HMB45^+^/CD8^+^ DNCs were identified in 93% (n = 26), with highly variable amounts ranging from 0 to 25 per 10 HPFs. Due to the knowledge that hybrid cells are present in the blood of patients with this disease, the fluctuation in numbers could either be due to these cells having already migrated to the blood from the primary tumor or because formation has not yet occurred [34]. Given that no statistically significant relationship was observed between these hybrid cells and the analyzed HRFs, and in conjunction with the positive correlation between the number of CD8^+^ T-cells and the number of HMB45^+^/CD8^+^ DNCs observed, these findings could be an indication that cytotoxic T-cell suppression may be involved in the occurrence of this event. One potential avenue of T-cell suppression is through T-regulatory (Treg) cells. An association has been demonstrated in the literature between the presence of Treg cells in cyclooxygenase-2-positive tumors; however, no direct relationship between the number of Tregs and survival has been shown [49,50]. When looking at the immune profile of UM tumors, CD8 T-cells represent the majority of the infiltration, with few CD4 or Treg cells present [49,51]. Nonetheless, these cells may play a crucial role in hybrid formation through the creation of an immune-suppressive environment. It is also important to note that the T-cell CD3-zeta chain is required for signal transduction, and some studies have shown that some T-cells in UM fail to differentiate or lack this zeta chain [52,53,54]. The inability to become activated, which is essential for an immune response (thus, the activation of cytotoxic functions in a subset of the T-cell population), may be a reason for T-cells being a predominant partner in this DNC formation. It is also possible that T-cells capable of being activated that are not exhausted or suppressed may still be able to participate in hybridization. More mechanistic studies are warranted to clarify this proposition.

Macrophages, which are part of the innate immune system and play a role in the inflammatory response, have been demonstrated to represent a worse patient prognosis in the context of UM, as opposed to other neoplasms, where the inflammatory response is usually favorable [20,21]. It is important to note that the eye is an immune-privileged site without lymphatics, which may explain this contradiction to other malignancies [55]. Tumor-associated macrophages (TAMs) are divided into the M1 and M2 subtypes. The former has been said to have pro-inflammatory or anti-tumor characteristics, whereas the latter promotes tumor progression through a reduction in T-cell responsiveness (i.e., immunosuppression) and the promotion of angiogenesis [56]. In UM, the M2 (CD68^+^/CD163^+^) population in particular has been shown to represent the majority of these macrophages, with some studies finding as many as 83% of the tumors to be positive for these cells, with moderate to high numbers [57,58]. Although we found the majority of our tumors to be positive for CD68-labeled macrophages (n = 23/24), 67% (n = 16/24) had a very low level of infiltration (<50 in 10 HPFs). This discrepancy, however, could be due to the heterogeneity of macrophages in UM tumors. A recent study characterized four different subtypes of macrophages in the context of this disease, which were present in different proportions for tumors with different classes of gene expression profiles [59]. Despite not finding moderate to high numbers of macrophages in the majority of our patient tumors, their potential role in DNC formation was still investigated, as the literature has shown macrophage–cancer cell fusion in breast and intestinal cancers, hinting that this cell type may still contribute to DNC formation [43,44]. In our study, HMB45^+^/CD68^+^ DNCs were identified in 71% (n = 17/24) of the tumors, and the quantity of these DNCs was found to positively correlate (*p* < 0.0001) with the level of TAM infiltration. When looking at the relationship between the presence of these hybrids and HRFs, significance was observed only with cell type following the Chi-squared test but not Fisher’s exact test. Given the small N-value for the number of tumors absent for DNCs (n = 7) and that this value is then split between the two possible cell types (i.e., epithelioid vs. spindle), the Chi-squared test, which calculates an approximate P value, may not have been accurate enough. Since Fisher’s exact test did not reveal this same significance, the result has been interpreted as not significant. Altogether, these results lead us to believe that macrophages do participate in DNC formation; however, the specific subtypes and their potential role after dissemination to the blood remains unclear. 

Cell–cell fusion is still not a fully understood or characterized process. It is a tightly regulated and energy-dependent process where the plasma membranes of cells under favorable conditions fuse, leading to the formation of a hybrid cell [36,37]. During a transitional state, hybrid cells carry two or more nuclei from different parental cells [36,37]. This is followed by a post-hybrid selection process to form a new, functional, altered hybrid cell [36,37]. Several studies have been published indicating that cell–cell fusion occurs in cancer, and that hybrid cells might include homotypic fusion between cancer cells or occur with normal cells from the microenvironment via heterotypic fusion [36]. In the context of normal melanocytes, exogenous (e.g., ultraviolet damage) or endogenous (e.g., oncogenic mutation) stressors as well as the development of a senescent phenotype can lead to the formation of multinucleated melanocytes [60,61]. As the literature suggests, our single cultures of NCMs did not reveal any polyploid cells. However, our results did demonstrate the presence of multinucleated melanocytes in the co-cultures, suggesting that the presence of PBMCs may either damage the NCMs or provide environmental stimuli, thus allowing for NCMs to multinucleate with one another (Figure 3). Moreover, normal melanocytes have also been shown to be capable of forming hybrids with melanoma cancer cells [62]. However, to the best of our knowledge, this hybridization has not been previously demonstrated between NCMs and PBMCs. Following these co-cultures, we did not observe the presence of hybrid cells but rather the presence of cell–cell interactions. In other words, this process of multinucleation appears to be a damage-induced or cancer-acquired trait. It is also important to note that the previous literature has not reported the presence of NCMs in the blood [30,63]. Therefore, these cells and their potential crosstalk with immune cells may be more relevant in the context of tumor initiation rather than its metastasis. 

To expand on the in vitro context of cell–cell fusion, the 92.1 UM cell line used in the in vitro studies was derived from different areas of a primary tumor that had 2–3+ infiltrating macrophages and 1–2+ infiltrating CD3 cells [41]. This cell line conserves the same mutation in GNAQ Q209L mutation, the gene responsible for encoding the alpha subunit of a heterotrimeric G protein that controls the activation of various downstream signaling pathways [64,65]. The Mel270 and OMM2.5 cell lines conserve a mutation in GNAQ *Q209P*, and they were derived from the primary site and liver metastasis of the same patient, respectively [65]. In this study, we found that co-culturing 92.1, Mel270, and OMM2.5 UM cells with PBMCs demonstrated the presence of a spontaneous cell–cell interaction, representing the formation of bi- or multinucleated cells after incubation (Figure 3). The formation of these double-labeled cells may involve the attachment of the PBMC and the malignant melanocyte, followed by a subsequent fusion process, thus creating a heterokaryon if bi-nucleated or a synkaryon if multinucleated [66]. The polyploid cells observed in the 92.1 UM cell line did not appear to undergo the process of genetic hybridization, which would result in a single nucleus being present. The reason for this may be that the cells in culture needed more time or different conditions to allow this increased ploidy to be resolved. On the other hand, the increased ploidy may be beneficial for hybrid cells. In fact, it has been suggested that polyploid cells may harbor an advantage against normal cells under stressful situations, such as chemotherapy or radiation, which are common therapeutic strategies against cancers [66,67,68,69]. Despite this, in the primary tumors, the majority of cells that fit the inclusion criteria set to be considered a DNC appeared to have only one nucleus, which is similar to what was observed in vitro with the Mel270 and OMM2.5 cell lines. However, some cells in the primary tumor representing a multinucleated population were identified and were similar to what was observed in the 92.1 UM cell line in vitro co-culture experiments. It is important to note that other studies on hybrid cells in various cancers do support the idea of fusion through the discovery of novel sets of RNA transcripts, the incorporation of genetic material, and patterns of gene expression [44,70,71,72]. In addition to this, hybrid cells have also been demonstrated in studies through the existence of tumor DNA in non-cancer cells or the presence of the Y chromosome in female cancer patients with a former bone marrow transplantation [73]. Further analyses are necessary to confirm that fusion between both cell types is the reason for DNC formation in this situation. Moreover, the presence of hybrid cells in vitro in both the primary (Mel270) and metastatic (OMM2.5) cell lines derived from a single patient suggests that these hybrids may in fact play a role in cancer progression and the development of metastasis. Further in vivo analyses would be required to draw specific conclusions about DNC involvement in metastasis. Nonetheless, the generation of DNCs in vitro might help in the study of the behavior of these cells and their use in platforms testing for novel UM therapeutics.

## 5. Conclusions

UM remains a disease with an unclear pathogenesis of systemic disease and metastasis. In this study, DNCs were identified in the majority of primary UMs tumors when double-labeled with HMB45 and the pan-leukocytic marker CD45. The discovery of this hybrid cell in the majority of the primary tumors suggests that these cells may play a role in the pathogenesis of the disease. When investigating leukocytic cell types capable of DNC formation, both CD68^+^ macrophages and CD8^+^ T-cells were identified as being able to participate in this event of hybrid cell formation. All of the primary melanomas in this study displayed T-cell infiltration, and the majority of them showed the presence of macrophages. Poor prognosis for patients with UM is associated with T-cell infiltration and a high density of macrophages; therefore, the presence of DNC formation with both cell types in primary UM suggests that this event may be a prominent mediator in the pathogenesis of the disease. The identification of the cell types capable of forming these novel DNCs creates a possible new therapeutic target. In addition to this, our studies revealed that UM cells retain the capacity to form DNCs in vitro in both primary (92.1 and Mel270) and metastatic (OMM2.5) cell lines, which may be the result of a heterotypic fusion.

We believe that this study will open new avenues to understanding UM development and progression. Future directions should investigate spontaneous DNC formation in vitro with other UM cell lines and PBMCs, including, in particular, CD8 T-cells and macrophages, to determine whether DNCs acquire any metastatic or growth advantages compared to the respective cancer cells. These in vitro co-cultures should also be expanded to include fluorescence in situ hybridization to assess for any genomic alterations (i.e., gain of function mutations) as a result of fusion. We also plan to study the tumorigenic capacity of DNCs in animal models to determine the kinetics of DNC genesis and to investigate whether drugs on the market or currently in clinical trials are able to target these cells for destruction to hopefully extend the period of progression-free survival for patients. In addition, upcoming studies should address the presence of DNCs at metastatic sites and assess the immune cell types involved in this hybrid cell formation. Finally, we plan to investigate the presence of HMB45^+^/CD68^+^ and HMB45^+^/CD8^+^ DNCs in the blood of UM patients and perform spatial transcriptomics on primary tumors to hopefully help shed more light on the pathogenesis of this disease.

## Figures and Tables

**Table 1:** UM patient characteristics.

Patient	Age	Sex	Location	Size	Cell Type	Vascular Loops	Mitotic Figures	Scleral Invasion
1	63	M	Iris—CB	Small	Mixed—SP	No	Yes	No
2	N/A	M	Choroid	Small	Mixed—EP	No	Yes	Yes
3	81	F	Choroid	Small	Mixed—EP	No	No	No
4	51	M	Choroid	Large	Epithelioid	No	No	No
5	76	F	Choroid	Large	Mixed—EP	Yes	Yes	No
6	76	F	Choroid	Large	Mixed—SP	No	No	No
7	63	F	Choroid	Large	Mixed—EP	No	Yes	No
8	55	M	Choroid	Large	Epithelioid	No	Yes	No
9	65	M	Choroid	Large	Mixed—EP	No	Yes	Yes
10	72	F	Choroid	Large	Spindle	No	Yes	Yes
11	N/A	N/A	Choroid	Small	Mixed—EP	No	No	Yes
12	81	M	Choroid	Large	Epithelioid	No	No	No
13	N/A	N/A	Choroid	Large	Epithelioid	No	Yes	Yes
14	76	M	Choroid	Large	Mixed—EP	No	No	No
15	76	M	Choroid—CB	Large	Epithelioid	No	No	No
16	59	F	Choroid	Small	Mixed—SP	Yes	Yes	No
17	57	M	Choroid—CB	Large	Epithelioid	Yes	No	Yes
18	77	M	Choroid	Large	Mixed—EP	No	No	No
19	50	M	Choroid	Small	Mixed—SP	No	No	Yes
20	71	M	Choroid	Small	Mixed—SP	No	No	No
21	62	F	Choroid	Large	Epithelioid	Yes	No	No
22	58	F	Choroid	Small	Epithelioid	No	No	No
23	50	F	Choroid	Large	Spindle	Yes	No	No
24	N/A	F	Choroid	Large	Epithelioid	Yes	No	No
25	67	F	Choroid—CB	Small	Mixed—SP	Yes	No	No
26	57	M	Choroid	Small	Mixed—SP	No	No	No
27	79	F	Choroid	Small	Mixed—EP	No	Yes	Yes
28	93	M	Choroid	Small	Mixed—EP	Yes	Yes	Yes
29	93	F	Choroid	Small	Mixed—SP	No	No	No
30	74	M	Choroid	Small	Mixed—EP	No	No	No
31	51	F	Choroid	Small	Mixed—SP	No	No	No
32	56	F	Choroid	Small	Mixed—EP	Yes	Yes	No
33	34	M	Choroid	Large	Mixed—SP	Yes	Yes	No
34	62	M	Choroid	Large	Mixed—SP	No	Yes	No
35	65	M	Choroid	Small	Mixed—EP	Yes	Yes	Yes
36	60	F	Choroid	Large	Mixed—SP	No	No	Yes
37	78	M	Choroid	Small	Epithelioid	Yes	Yes	No
38	47	M	Choroid	Large	Mixed—SP	No	No	No

N/A: not available; M: male; F: female; CB: ciliary body; EP: epithelioid-predominant; SP: spindle-predominant.

**Table 2:** Association between histopathologically determined HRFs and the presence or absence of DNCs.

DNC Staining	Size		Cell Type		Vascular Loops		Mitotic Figures		Scleral Invasion	
	X^2^	FET	X^2^	FET	X^2^	FET	X^2^	FET	X^2^	FET
HMB45/CD45 (n = 29)	0.0583	0.0996	0.6725	>0.9999	0.2155	0.2668	0.5844	>0.9999	0.2189	0.5320
HMB45/CD8 (n = 28)	>0.9999	>0.9999	0.8322	>0.9999	0.2378	0.5053	0.9136	>0.9999	0.3968	0.4444
HMB45/CD68 (n = 24)	0.6534	>0.9999	0.0247 *	0.0686	0.5254	0.6466	0.0577	0.0850	0.6123	>0.9999

X^2^: Chi-squared test; FET: Fisher’s exact test; * statistically significant at *p* < 0.05.

## Data Availability

The original contributions presented in the study are included in the article/supplementary material, further inquiries can be directed to the corresponding author.

## Supplementary Materials

The following supporting information can be downloaded at https://www.mdpi.com/article/10.3390/cancers16183231/s1, Figure S1: Representative images of positive control special stains in UM at 10 × magnification; Figure S2: Representative images of UM lesions with histopathological high-risk factors in UM; Figure S3: Representative images of the levels of infiltration of inflammatory cells in primary UM tumors at 10× magnification; Figure S4: Multinucleated HMB45^+^/CD8^+^ DNCs; Figure S5: NCMs and PBMCs alone and their co-cultures; Figure S6: Multinucleated cell formation following co-culture experiments.
