# Supplementary material for: The Phenotypical Characterization of Dual-Nature Hybrid Cells in Uveal Melanoma
Source: Cancers (Basel). 2024 Sep 22;16(18):3231. doi: 10.3390/cancers16183231 (PMC11429545; doi:10.3390/cancers16183231)
Supplement: Supplementary file 1 [file cancers-16-03231-s001.zip › Supplemental Figures.pdf]

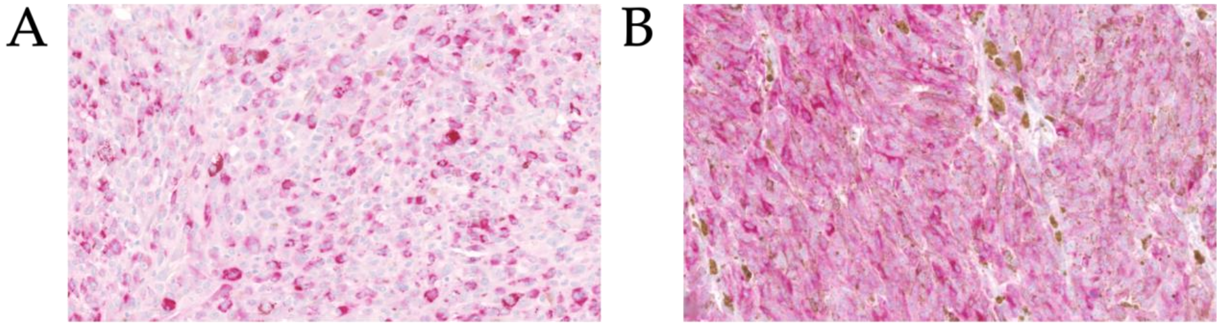

Figure S1. Representative images of positive control special stains in UM at 10X magnification. (**a**) HMB45 positive (red). (**b**) Melan-A positive (red).

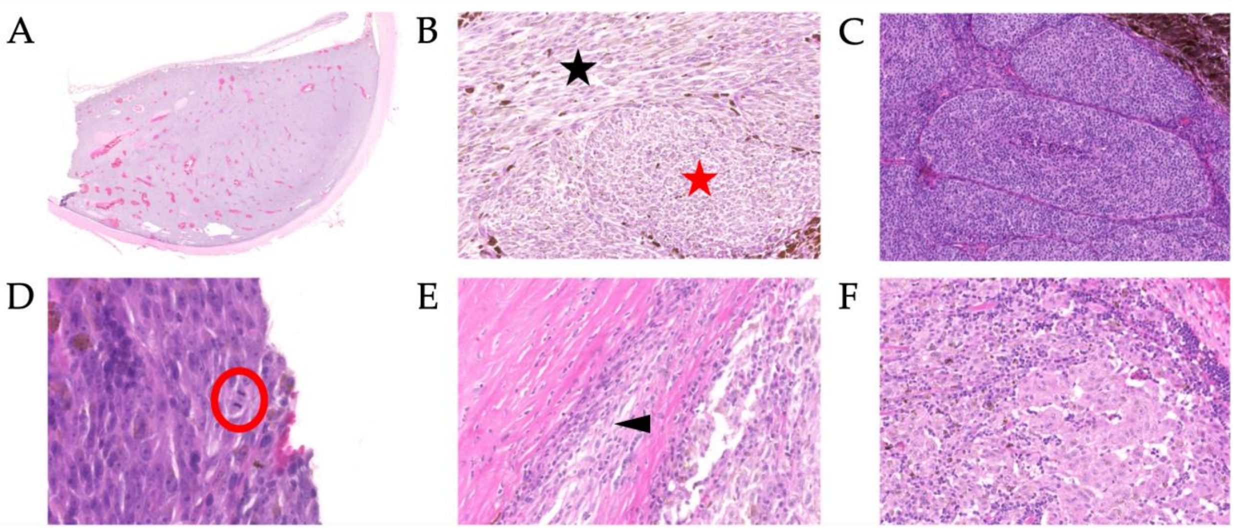

Figure S2. Representative images of UM lesions with histopathological high-risk factors in UM. (**a**) Posterior eye section view of a large tumor. (**b**) Mixed cell tumor with spindle cells (black star) and epithelioid cells (red star). (**c**) Tumor vascular closed loops. (**d**) Mitotic figure (red circle). (**e**) Tumor with scleral invasion (black arrow). (**f**) Tumor with lymphocytic infiltration.

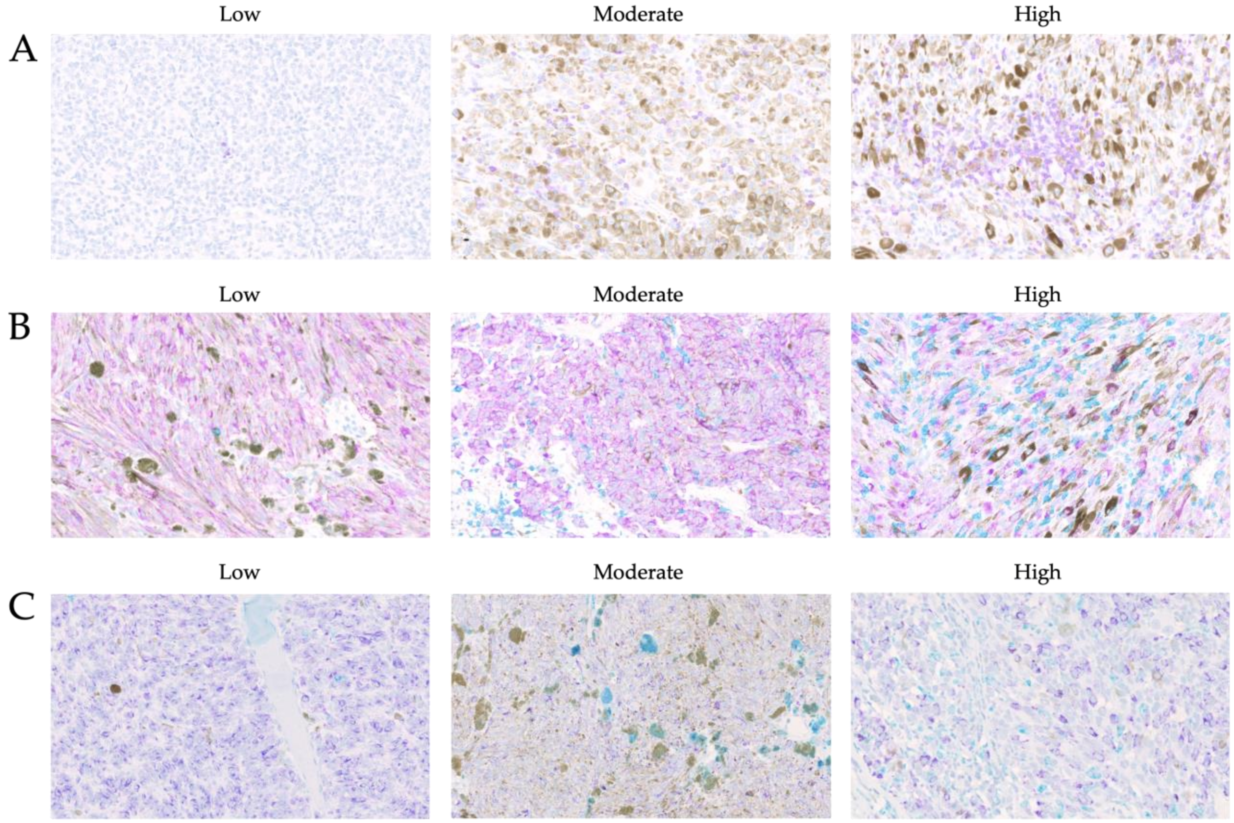

Figure S3. Representative images of the levels of infiltration of inflammatory cells in primary UM tumors at 10X magnification. (**a**) Low, moderate and high count of CD3$^+$ T-cells (red). (**b**) Low, moderate and high count of CD8$^+$ T-cells (teal). (**c**) Low, moderate and high count of CD68$^+$ macrophages (teal).

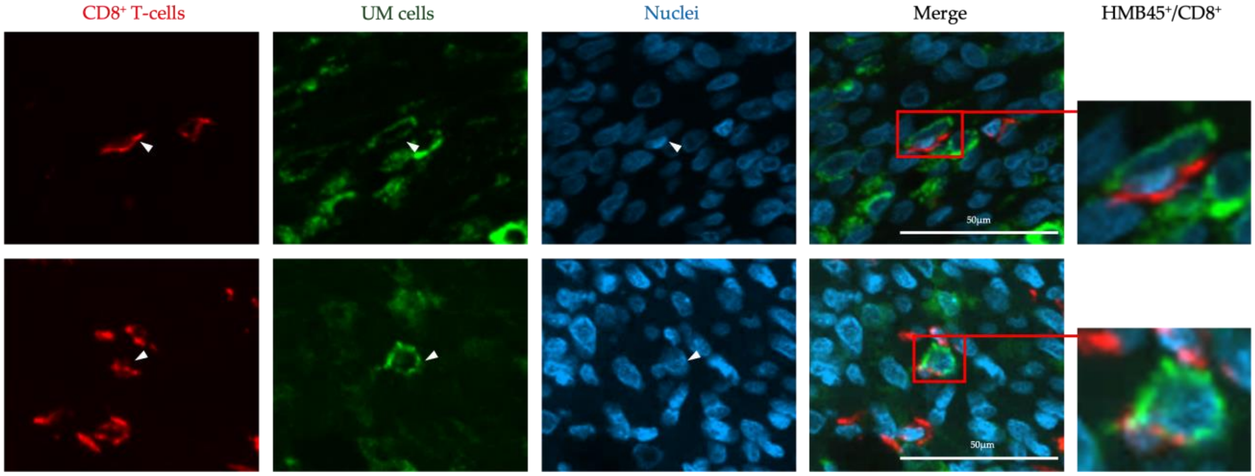

Figure S4. Multinucleated HMB45$^+$/CD8$^+$ DNCs. Immunofluorescent labelling with CD8, HMB45, and DAPI (nuclei) of multinucleated HMB45$^+$/CD8$^+$ DNCs (merge; white arrowheads) and their higher power images on the right.

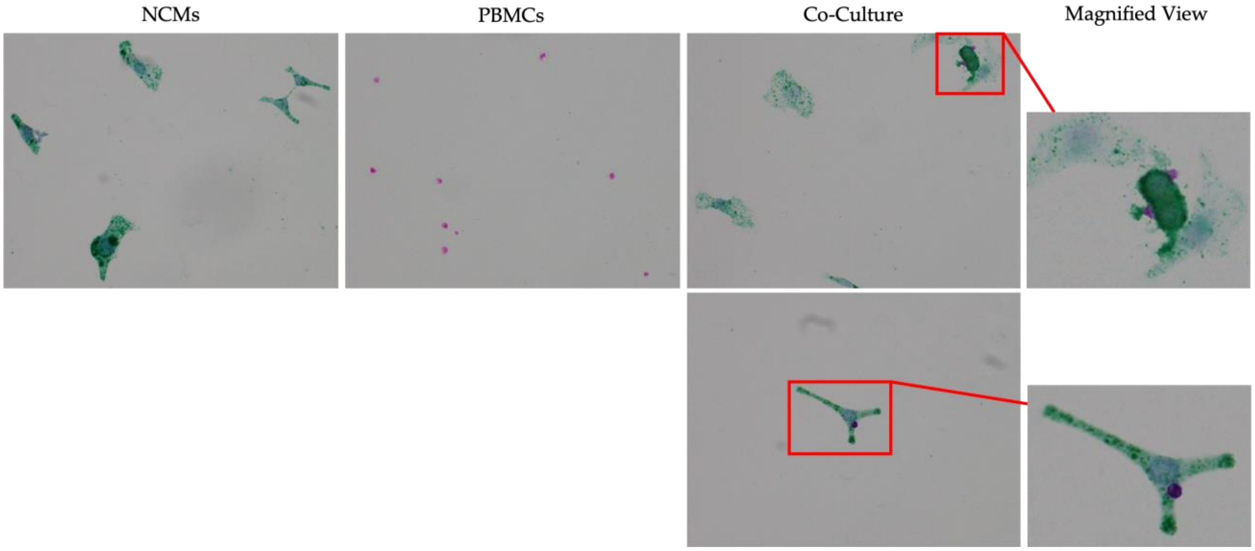

Figure S5. NCMs and PBMCs alone and their co-cultures. Cells were imaged at 40X. Single stain of NCMs (green), PBMCs (purple), and co-cultures revealing a NCM and PBMC interaction (red box) as well as a higher power image of the interaction.

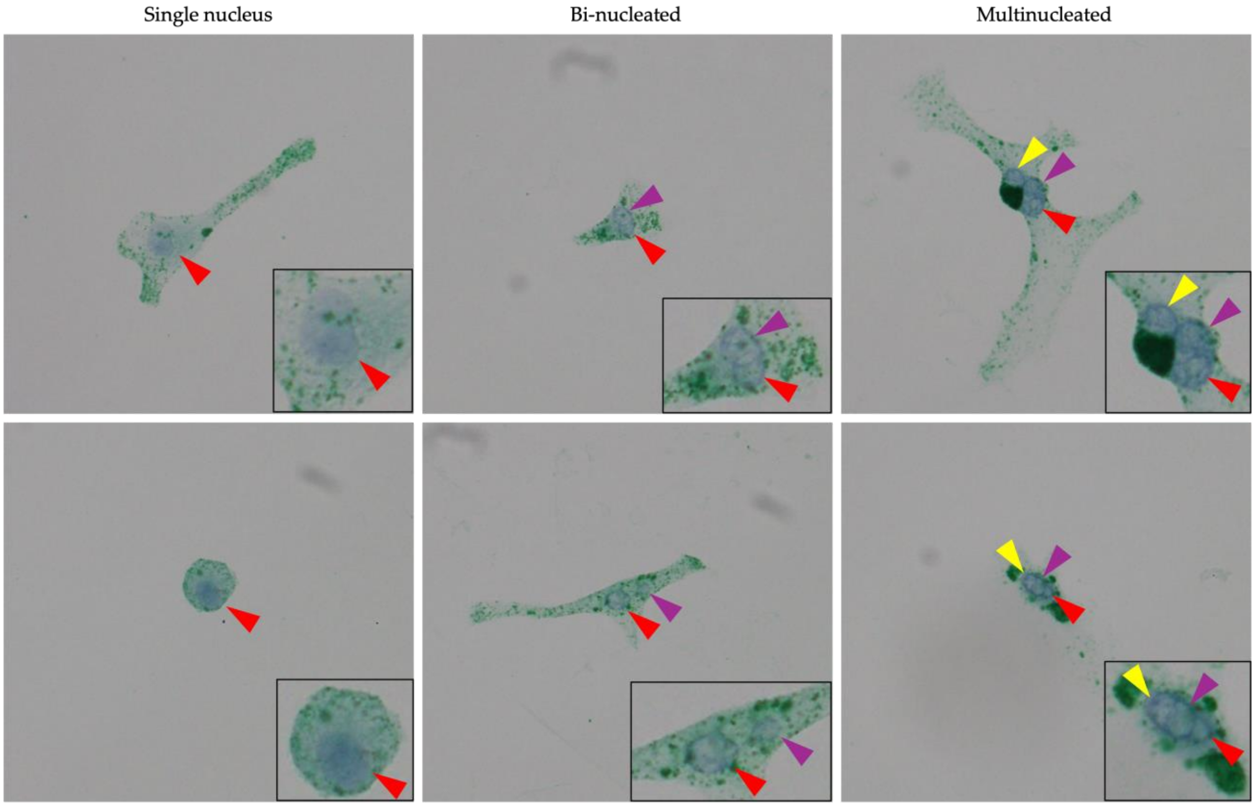

Figure S6. Multinucleated cell formation following co-culture experiments. NCM cells imaged at 40X in co-cultures. Single, bi- and multinucleated NCMs (red, purple, and yellow arrows respectively) were observed in co-cultures. Magnified views of these nuclei are shown in the bottom right corner of the respective images.
